# Dissociation of Increases in PGC-1α and Its Regulators from Exercise Intensity and Muscle Activation Following Acute Exercise

**DOI:** 10.1371/journal.pone.0071623

**Published:** 2013-08-12

**Authors:** Brittany A. Edgett, William S. Foster, Paul B. Hankinson, Craig A. Simpson, Jonathan P. Little, Ryan B. Graham, Brendon J. Gurd

**Affiliations:** 1 School of Kinesiology and Health Studies, Queen’s University, Kingston, Ontario, Canada; 2 Department of Emergency Medicine, Queen’s University, Kingston, Ontario, Canada; 3 School of Health and Exercise Sciences, University of British Columbia Okanagan, Kelowna, British Columbia, Canada; 4 School of Physical and Health Education, Nipissing University, North Bay, Ontario, Canada; Mayo Clinic, United States of America

## Abstract

Muscle activation as well as changes in peroxisome proliferator-activated receptor gamma coactivator 1 alpha (PGC-1α) following high-intensity interval exercise (HIIE) were examined in young healthy men (n  = 8; age, 21.9±2.2 yrs; VO_2_peak, 53.1±6.4 ml/min/kg; peak work rate, 317±23.5 watts). On each of 3 visits HIIE was performed on a cycle ergometer at a target intensity of 73, 100, or 133% of peak work rate. Muscle biopsies were taken at rest and three hours after each exercise condition. Total work was not different between conditions (∼730 kJ) while average power output (73%, 237±21; 100%, 323±26; 133%, 384±35 watts) and EMG derived muscle activation (73%, 1262±605; 100%, 2089±737; 133%, 3029±1206 total integrated EMG per interval) increased in an intensity dependent fashion. PGC-1α mRNA was elevated after all three conditions (*p*<0.05), with a greater increase observed following the 100% condition (∼9 fold, *p*<0.05) compared to both the 73 and 133% conditions (∼4 fold). When expressed relative to muscle activation, the increase in PGC-1α mRNA for the 133% condition was less than that for the 73 and 100% conditions (*p*<0.05). SIRT1 mRNA was also elevated after all three conditions (∼1.4 fold, *p*<0.05), with no difference between conditions. These findings suggest that intensity-dependent increases in PGC-1α mRNA following submaximal exercise are largely due to increases in muscle recruitment. As well, the blunted response of PGC-1α mRNA expression following supramaximal exercise may indicate that signalling mediated activation of PGC-1α may also be blunted. We also indentify that increases in PDK4, SIRT1, and RIP140 mRNA following acute exercise are dissociated from exercise intensity and muscle activation, while increases in EGR1 are augmented with supramaximal HIIE (*p*<0.05).

## Introduction

Mitochondrial biogenesis is a hallmark of the adaptive response of skeletal muscle to periods of exercise training [1], [2]. Peroxisome proliferator-activated receptor gamma coactivator 1 alpha (PGC-1α) coordinates the activation of both nuclear and mitochondrial transcription factors and helps to regulate this adaptive response [3]. Following acute bouts of exercise, PGC-1α transcriptional activity is increased, resulting in elevated mRNA expression of both itself and a variety of mitochondrial genes [4], [5]. Sirtuin 1 (SIRT1) has been implicated in the control of PGC-1α in skeletal muscle via deacetylation [6], and changes in SIRT1 content have also been implicated in the induction of mitochondrial biogenesis that accompanies exercise training [7], [8]. Given the apparent relationship between PGC-1α, SIRT1 and chronic increases in mitochondrial proteins, understanding the impact of exercise intensity on PGC-1α and SIRT1 is of considerable interest.

Several of the intracellular signalling molecules involved in the activation of PGC-1α are activated in an intensity-dependent manner following submaximal exercise. Specifically, intensity dependent activation of both AMPK [9]–[11] and CAMK/p38 MAPK [10], [12] signalling have been observed. While SIRT1 activity following exercise of different intensities has not been directly measured, activation of SIRT1 by NAD^+^ availability (via AMPK) [13] is also expected to be intensity dependent. Consistent with changes in intracellular signalling events, several reports have demonstrated greater increases in PGC-1α mRNA expression following higher intensities of submaximal steady-state exercise in athletes [14], young sedentary adults [10] and patients with type II diabetes [15]. While these studies demonstrate intensity-dependent increases in PGC-1α mRNA following submaximal exercise, no study to date has attempted to establish whether the greater increases in PGC-1α mRNA observed following high-intensity submaximal exercise extend to supramaximal intensities.

Much less in known regarding the impact of exercise intensity on changes in SIRT1 mRNA expression and there is currently considerable controversy regarding changes in SIRT1 following exercise training [16], [17]. However, there is evidence that SIRT1 mRNA expression increases in skeletal muscle in response to mechanical stress in C2C12 cells and the diaphragm of mice [18], as well as in human skeletal muscle following extreme bouts of endurance exercise [19], [20]. At present, we are unaware of any studies that have examined the impact of exercise intensity on changes in SIRT1 mRNA expression following an acute bout of exercise.

Our current understanding of the mechanisms underlying intensity-dependent increases in both PGC-1α and SIRT1 is also obscured by methodological limitations imposed by the needle biopsy technique. Specifically, analysis of both intracellular signalling events and changes in gene expression in muscle biopsy homogenates represent an average response within all of the fibres sampled. Based on known differences in fibre type recruitment during different intensities of exercise [21], the greater increases in PGC-1α expression following high-intensity steady-state exercise may result from either a greater activation of PCG-1α within fast-twitch (Type II) fibres, similar activation of PGC-1α within a greater population of fibres, or a combination of both [22], [23]. Electromyography (EMG) provides a non-invasive, continuous, real-time analysis technique for quantification of muscle activation during dynamic exercise [24]. This technique has recently been combined with the muscle biopsy technique to normalize changes in enzyme activation to EMG derived estimates of muscle activity [25]. Using this integrative approach, measures of gene expression (from muscle biopsy samples) can be normalized to muscle activation (EMG) in order to provide insight into whether intensity dependent increases in PGC-1α (and potentially SIRT1) are a result of an intensity effect within individual muscle fibres, or simply a product of increased muscle/fibre recruitment.

The purposes of the current study were: 1) to determine if increases in PGC-1α induced by higher intensities of submaximal exercise extend to supramaximal exercise by comparing changes in gene expression following three intensities (submaximal, maximal, and supramaximal) of matched volume exercise, 2) to determine the impact of exercise intensity on changes in gene expression of regulators of PGC-1α activity, and 3) to determine if greater increases in PGC-1α gene expression at higher intensities can be explained by intensity-dependent increases in muscle activation. It was hypothesized that the increase in gene expression of both PGC-1α and SIRT1 would be greater at higher intensities of exercise, and that this increase in gene expression would be largely explained by intensity-dependent increases in muscle recruitment.

## Methods

### Experimental Approach

To study the acute effects of different intensities of exercise on mRNA expression of PGC-1α and its regulators, participants performed three separate sessions of high-intensity interval exercise (HIIE) targeting 73, 100, and 133% of their peak aerobic power. All interval sessions were matched for total work (kJ). mRNA expression was determined in muscle samples obtained before and three hours after each interval session. Muscle activation was continuously monitored during each interval of each session using surface EMG. EMG data was utilized to normalize changes in mRNA relative to muscle activation using previously published methodology [25].

### Participants

Lean healthy men (n  = 8) volunteered to participate in the present study. Participant characteristics are presented in Table 1. All participants were recreationally active but were not involved in a specific training program at the time of recruitment. The experimental protocol and associated risks were explained both orally and in writing to all participants before written consent was obtained. The study was approved by the Health Sciences Research Ethics Board at Queen’s University.

**Table 1:** Subject characteristics (n  = 8).

Age (yr)	21.9±2.2
Height (cm)	182±5
Body Mass (kg)	77.1±8.5
BMI (kg/m^2^)	23.2±1.6
VO_2_peak (ml/min/kg)	53.1±6.4
Peak WR (watts)	317±23.5

Values are presented as mean ± SD.

### VO_2_peak Test and HIIE Protocols

During an initial visit to the laboratory participants performed a VO_2_peak test on a cycle ergometer (Monark, Ergomedic 874E, Varberg, Sweden) and had anthropometric measures (height, weight, and waist circumference) recorded. The VO_2_peak test consisted of load-less pedaling for five minutes at 80 RPM, followed by a step increase to 80 watts for one minute. After the step increase to 80 watts, work rate was increased by 25 watts per minute until the participant reached volitional fatigue (determined by the inability of the participant to maintain a minimum cadence of 60 RPM). Gas exchange was collected throughout the test using a metabolic cart (Moxus, AEI Technologies, Pittsburgh, PA) and VO_2_peak was calculated using the highest 30 second average VO_2_ during the final stage of the ramp protocol. During the VO_2_peak test participants were familiarized with a restraint system intended to standardize leg position, maintain posture, and prevent participants from standing during the subsequent HIIE visits.

During the first visit, participants completed a dietary record for the 24 hours prior to arriving at the lab. Before every subsequent visit participants were given a copy of this record and were asked to consume the same meal for dinner the night before each interval session. For all experimental visits, participants were instructed to refrain from exercise for 24 hours prior to their visit, and arrived to the lab at 8:00 am after having fasted for 12 hours. Following a standardized breakfast (toasted bagel [∼190 kcal; 1 g fat, 36 g carbohydrate, 7 g protein] with 15 g of cream cheese [∼45 kcal; 4 g fat, 1 g carbohydrate, 1 g protein]), participants rested in a seated position for one hour after which the first of two muscle biopsies were obtained from the vastus lateralis muscle as close as possible to the recommended EMG placement locations based on SENIAM (Surface EMG for Non-Invasive Assessment of Muscles) [26], using the needle biopsy technique [27] with manual suction. Biopsies were taken through a small incision in the skin to the deep fascia under deep (2% lidocaine) and superficial (2% lidocaine with epinephrine) local anesthesia. Following this first biopsy, participants were moved to the cycle ergometer where EMG electrodes were applied over the vastus lateralis of the leg opposite to the one that had been biopsied at the predetermined location. Participants then performed HIIE at a target work rate (WR) of 77, 100, or 133% of their peak aerobic power (highest 30 second power output [watts] from VO_2_peak test). Following completion of HIIE participants rested in a seated position for three hours before a second muscle biopsy sample was taken from close proximity (approximately 10–20 mm) to the first biopsy on the same leg. Muscle biopsy samples were immediately frozen in liquid nitrogen and stored at minus 80°C until analysis.

Following a randomized crossover design, each participant performed three different HIIE protocols on a cycle ergometer (Monark, Ergomedic 874E, Varberg, Sweden) separated by a washout period of approximately one week (minimum of and maximum of 2 weeks). All interval protocols consisted of a five minute load-less warm up, followed by HIIE consisting of one minute intervals separated by one minute of loadless cycling at a cadence of the participants choosing on a cycle ergometer. During the intervals a load was added to the bike such that cycling at 80 rpm would result in a WR of 73, 100, or 133% of peak aerobic power. These intensities were chosen so that a matched amount of external work would be achieved in 11, 8 and 6 intervals for each intensity, respectively. Subjects were instructed to maintain 80 rpm at all times, however when rpm fell below 80, addition intervals were added such that the target amount of work (± ∼5%) was achieved in all HIIE sessions (See Table 2).

**Table 2:** Descriptive statistics for individual interval protocols.

Exercise Protocol	73%	100%	133%
Target Intervals	11	8	6
Intervals Performed	11.5±0.5	9.0±0*	7.2±0.4†
Mean Interval WR (watts)	237±21	323±26*	384±35†
Mean Interval Intensity (% of peak)	75±6	102±8*	121±11†
External Work (kJ)	731±38	752±59	702±60
Average Interval HR (bpm)	135±12	154±7*	162±7*

Values presented as mean ± SD.

* Significantly (p<0.05) different from 73%.

† Significantly (p<0.05) different from both 73% and 100%.

### EMG Procedures

Vastus lateralis muscle activity was monitored unilaterally using pre-gelled, self-adhesive, bipolar Ag/AgCl EMG electrodes (EasyTrode 3SG3-N, MultiBioSensors, El Paso, TX, USA). The electrodes were placed longitudinally with respect to the underlying muscle fibre arrangement, as per the recommendations of SENIAM [26]. Before the electrodes were applied, the skin was shaved and abraded with alcohol to minimize impedance, and then wires were well secured with adhesive tape to avoid movement-induced artefacts [28]. RPM was monitored using a magnetic switch with the components secured to the ergometer crankshaft and body.

Raw EMG signals were bandpass filtered (10–1000 Hz) and amplified (AMT-8, Bortec Biomedical Ltd., Calgary, AB, Canada; input impedance = 10 GΩ, CMRR = 115 dB at 60 Hz), and then captured digitally concurrent to the switch data at 2048 Hz using a USB-6008 A2D board and custom Labview software (National Instruments, Austin, TX, USA). To normalize the EMG signals obtained during testing to their amplitudes obtained during maximum voluntary contractions (MVC), a series of three seated MVC were performed prior to testing on each day.

### EMG Processing

Custom Matlab software (The MathWorks, Natick, MA, USA) was used for all data processing [29], [30]. After the DC bias was removed, the raw EMG data were bandpass filtered between 20–450 Hz. These data were then full-wave rectified and low-pass filtered using a 2^nd^ order dual-pass Butterworth filter with a 6 Hz cutoff frequency in order to produce a linear envelope. The linear enveloped data were then normalized to the peak activation value produced during the MVC trials. RPM was also calculated from the magnetic switch data; this was required to split each 60 second interval into individual pedal cycles, each with one primary muscular contraction.

To then quantify the amount of muscular activity under the different interval intensities, three methods were used: 1) after dividing the data into individual pedal cycles the peak value of each muscular contraction was taken, and then the average of the peaks was first taken across each interval, and then across each interval within a certain condition to obtain one average peak value per participant per intensity condition; 2) to get an idea of the total activity within each 60 second interval, the integrated EMG (iEMG) (the total area under the curve) was taken and then averaged across each interval within a certain condition; and 3) to get an idea of the overall amount of muscular activity across all intervals under a certain training intensity, the total area under the curve was taken across each 60 second interval, and then all intervals were summed together.

### Determination of Gene Expression

RNA was extracted using a modified version of the single-step method by guanidinium thiocyanate-phenol-chloroform extraction [31]. The purified RNA pellet was dissolved in RNase and DNase-free ultrapure water then quantified spectrophotometrically at 260 nm. Protein contamination was assessed by measuring absorbance at 280 nm. Samples had an average 260:280 ratio of 2.01±0.10 (mean ± SD). One microgram of resulting RNA was reverse transcribed using the QuantiTect Reverse Transcription Kit (Qiagen, Mississauga, Canada). Transcript levels were determined on an ABI 7500 Real Time PCR System (Foster City, CA) using the following protocol: 1 cycle at 95°C for 15 minutes, 40 cycles of 95°C for 15 seconds, 30 seconds at the primer set specific annealing temperature (Table 3), and 72°C for 36 seconds, followed by a dissociation curve to assess specificity of the reaction. Primer set efficiencies were determined using real-time PCR with an appropriate cDNA dilution series prior to sample analysis. Average primer set-specific efficiencies [32] were *E* **= **2.05±0.06 (mean ± SD). All samples were run in duplicate 25 µl reactions containing: 50 ng cDNA, 0.58 µM primers, and GoTaq PCR Master Mix (Promega, Madison, WI). No-template controls were run with water in place of cDNA to ensure the absence of contamination. Results were analyzed according to the *Δ*C_t_ method using TATA-binding protein (TBP) as a housekeeping gene [33]. Primer sets are also listed in Table 3.

**Table 3:** List of primer sequences used for real-time PCR and their specific annealing temperatures.

Gene	Forward Primer (5'-3')	Reverse Primer (5'-3')	T (°C)	NCBI RefSeq
PGC-1α	CACTTACAAGCCAAACCAACAACT	CAATAGTCTTGTTCTCAAATGGGGA	59	NM_013261.3
PDK4	CGGCTGGTGGGAAGACTTGA	TGCCGCGGAGTGAAGAGTCT	59	NM_002612.3
SIRT1	AGAACATAGACACGCTGGAACA	CAAGATGCTGTTGCAAAGGAACC	59	NM_012238.4
GCN5	AAGGACCCCGACCAGCTCTA	GGGAAGCGGATGACCTCGTA	59	NM_021078.2
RIP140	GTTCCACTCAGCCCAGCAGT	AGACCCTGCACAGCCCAAGT	59	NM_003489.3
EGR1	AGCAGCCCTACGAGCACCT	CGAGTGGTTTGGCTGGGGTA	59	NM_001964.2
p53	CCAACAACACCAGCTCCTCT	CCTCATTCAGCTCTCGGAAC	61	NM_000546.5
TBP	AGACGAGTTCCAGCGCAAGG	GCGTAAGGTGGCAGGCTGTT	59	NM_003194.4

(T).

See text for definitions of abbreviations.

### Statistical Analysis

Differences between interval protocols, and changes in EMG derived markers of muscle activation were assessed using one-way repeated measures ANOVA. The effect of HIIE intensity on gene expression was assessed via two-way repeated measures ANOVA. Statistical analysis of gene expression was performed on linear data using the *Δ*C_t_ method [33]. Any significant main effects or interactions were subsequently analyzed using a Bonferroni post-hoc test where appropriate. Statistical significance was accepted at *p*<0.05, unless otherwise noted.

## Results

### Characteristics of HIIE

Characteristics for all exercise protocols are presented in Table 2. Consistent with the study design, mean interval WR, mean interval intensity and average interval HR were all lowest in the 73% condition and greatest in the 133% condition. External work performed was not statistically different between conditions.

### Muscle Activation

**Figure 1:**
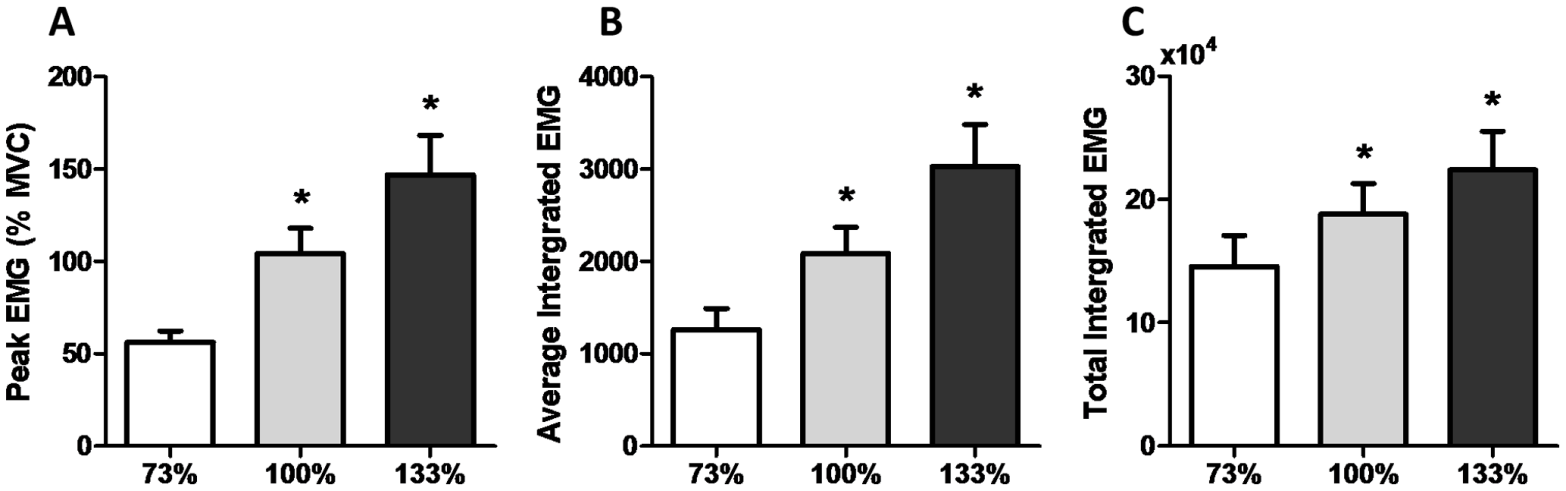
EMG derived estimates of muscle activation during high-intensity interval exercise. Muscle activation, assessed by average peak pedal stroke EMG signal (A), integrated EMG signal averaged per 60 second interval (B), and total integrated EMG signal (C). * Significantly (*p*<0.05) different from 73%.

EMG derived estimates of muscle activation in the vastus lateralis during HIIE are presented in Figure 1. Peak EMG (expressed as % MVC) was significantly higher in the 100 and 133% conditions compared to the 73% condition (*p*<0.05, Figure 1A). Average integrated EMG per interval was also significantly higher in the 100 and 133% conditions (*p*<0.05, Figure 1B), as was the total integrative EMG (*p*<0.05, Figure 1C).

### Gene Expression

Tata binding protein (TBP) was stable across all conditions with no difference in the raw CT values observed between any time point or condition (73%, pre 25.68±0.49, post 25.54±0.15; 100%, pre 25.26±0.53, post 24.94±0.32; 133%, pre 25.28±0.70, post 25.64±0.67).

**Figure 2:**
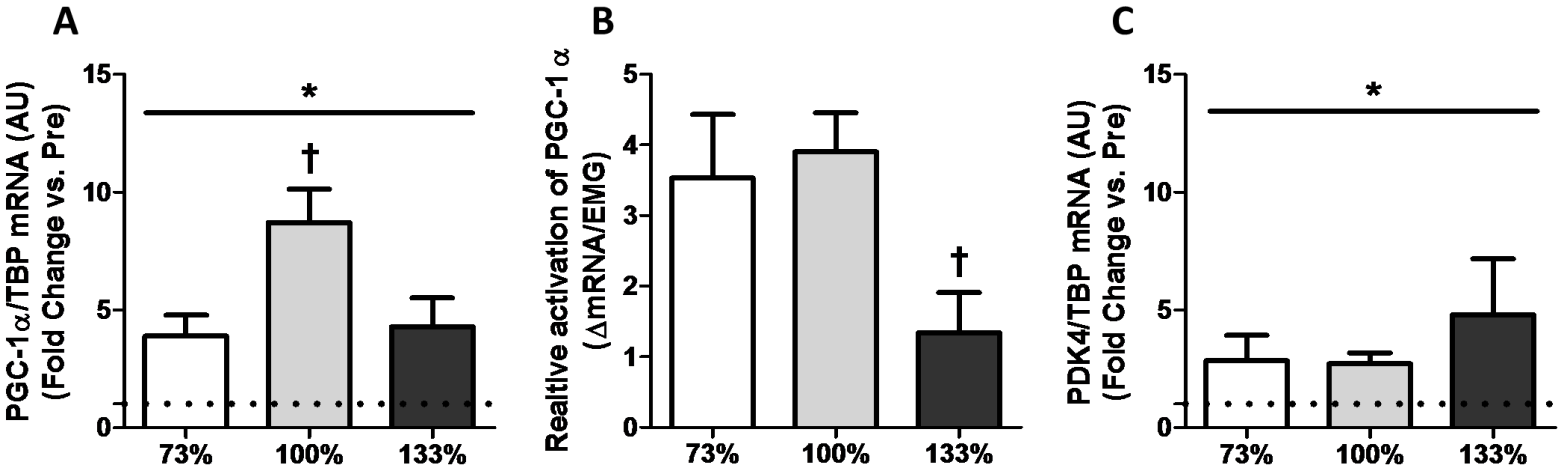
Impact of interval intensity and muscle activation on the expression of PGC-1α and PDK4. Impact of (A) interval intensity and (B) muscle activation on the expression of PGC-1α and (C) PDK4. The change in mRNA expression from baseline to post-exercise for interval intensities of 73% (open bars), 100% (grey bars) and 133% (dark bars) is shown. Values presented as Mean ± SEM. * Significant (*p*<0.05) main effect of time. † Significantly (*p*<0.05) different than other exercise intensities.

PGC-1α mRNA expression increased following all HIIE conditions (*p*<0.05, Figure 2). Interestingly, the 100% condition (+790%) increased significantly more than both the 73 (+432%) and 133% (+424%) conditions (*p*<0.05, Figure 2A). When expressed relative to EMG derived estimates of muscle activation, (peak, average/interval, and cumulative), PGC-1α gene expression was significantly lower in the 133% condition compared to both the 73 and 100% conditions (*p*<0.05, Figure 2B). Conversely, mRNA expression of the downstream PGC-1 α target [34], pyruvate dehydrogenase kinase isozyme 4 (PDK4), had a significant main effect of time with exercise, however no differences were observed between intensity conditions (*p*<0.05, Figure 2C).

**Figure 3:**
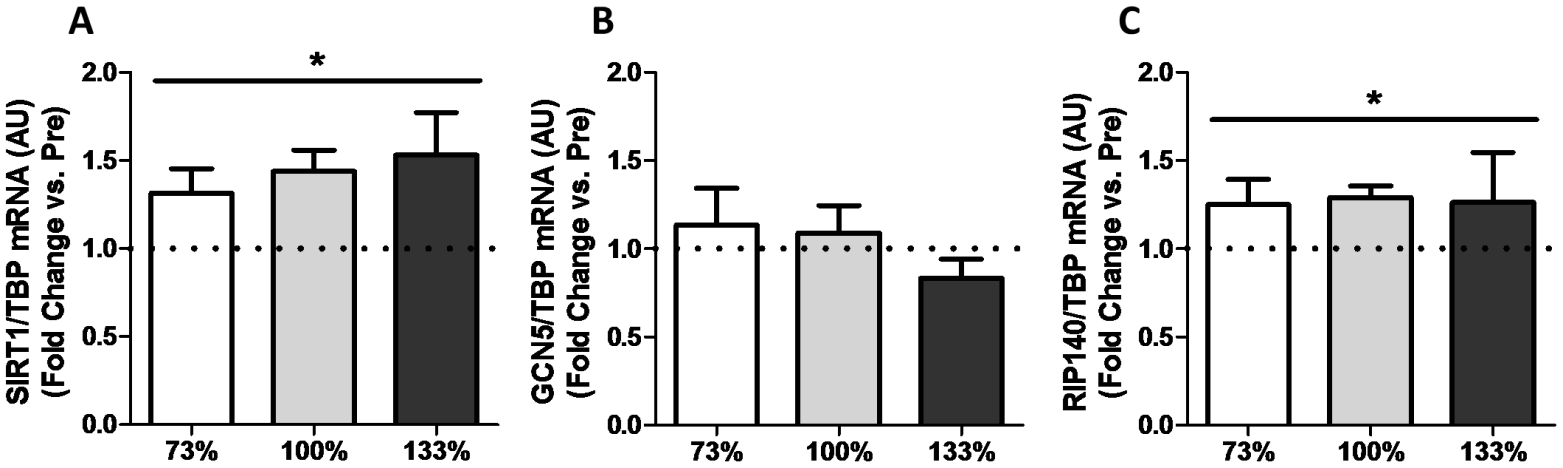
Impact of interval intensity on the expression of PGC-1α regulators. Impact of interval intensity on the expression of (A) SIRT1, (B) GCN5, and (C) RIP140. The change in mRNA expression from baseline to post-exercise for interval intensities of 73% (open bars), 100% (grey bars) and 133% (dark bars) is shown (A). Values presented as Mean ± SEM. * Significant (*p*<0.05) main effect of time.

**Figure 4:**
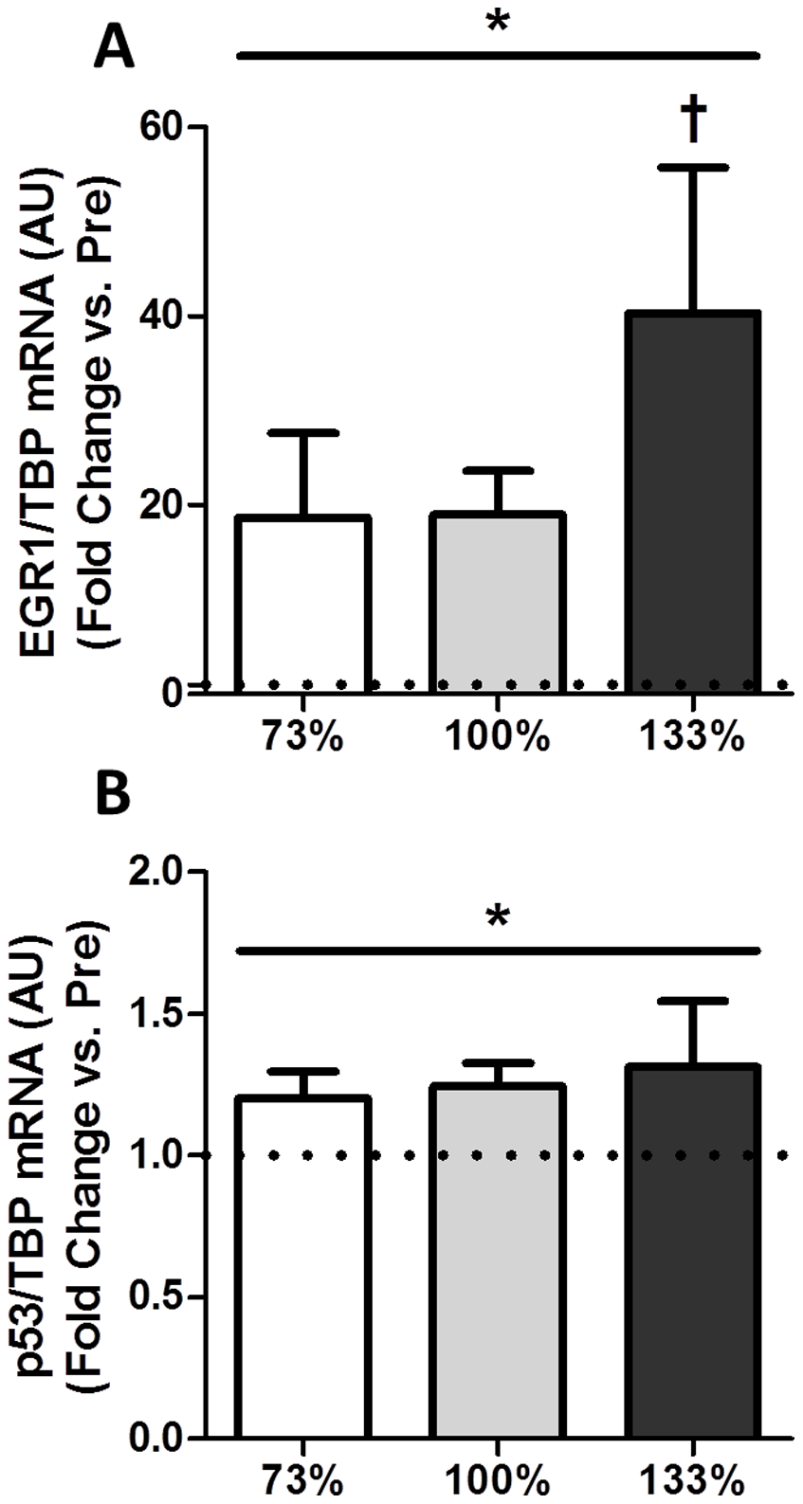
Impact of interval intensity on the expression of EGR1 and p53. Impact of interval intensity on the expression of (A) EGR1 and (B) p53. The change in mRNA expression from baseline to post-exercise for interval intensities of 73% (open bars), 100% (grey bars) and 133% (dark bars) is shown (A). Values presented as Mean ± SEM. * Significant (*p*<0.05) main effect of time. † Significantly (*p*<0.05) different than other exercise intensities.

Upstream of PGC-1α, SIRT1 mRNA expression also increased following all three intensities of HIIE (*p*<0.05), with no effect of intensity observed (Figure 3A). Relative to EMG signals, the increase in SIRT1 was greater in the 73% than both the 100 and 133% conditions (data not shown). Expression of the acetyltransferase general control non-repressible 5 (GCN5), a negative regulator of PGC-1α activity [35], did not change in response to any exercise intensity (Figure 3B). Gene expression of another negative regulator of PGC-1α activity, receptor interacting protein 1 (RIP140), also demonstrated a significant main effect of time following HIIE (*p*<0.05, Figure 3C). Gene expression of tumor protein 53 (p53) and early growth response 1 (EGR1) both demonstrated a significant main effect of time (*p*<0.05, Figure 4), with a greater increase in EGR1 observed following supramaximal HIIE (*p*<0.05).

## Discussion

The current study compared changes in PGC-1α and SIRT1 mRNA following matched volume exercise of three different intensities. We were able to successfully match total exercise volume across conditions while increasing the average exercise intensity as per our study design (Table 2). Consistent with the greater intensities of exercise elicited, there was an intensity-dependent increase in EMG estimates of muscle activation (Figure 1). Our major findings were: 1) consistent with previous reports [10], [14], [15] a greater increase in PGC-1α mRNA expression was observed following maximal (100%) compared to submaximal (73%) exercise, 2) surprisingly, this intensity effect did not extend to supramaximal exercise where the increase in PGC-1α mRNA was similar to that observed following submaximal exercise (73%) and less than that observed following maximal exercise (100%), 3) when expressed relative to muscle activation, the greater increase in PGC-1α following 100% compared to 73% of peak aerobic power appears to be primarily a result of increased muscle recruitment, and 4) PDK4, SIRT1, and RIP140 mRNA expression were elevated following all intensities of exercise; however, this increase was similar between conditions, suggesting that the mechanisms underlying these responses are not intensity-dependent.

### Exercise Intensity and Increases in PGC-1α Expression

Previous reports comparing two submaximal exercise intensities have demonstrated greater increases in PGC-1α mRNA following higher versus lower intensities of exercise [10], [14], [15]. Our finding of a greater increase in PGC-1α mRNA following 100%, compared to 73% is consistent with these reports; however, this apparent intensity effect did not extend to supramaximal exercise (Figure 2). Further, PDK 4 which is both a target of PGC-1α co-activation [34] and has previously been observed to increase to a greater extent following higher intensities of exercise [36], increased equally following all intensities of exercise. These results, combined with the association observed between increases in PGC-1α mRNA and protein and the induction of mitochondrial biogenesis during exercise training [5], question whether supramaximal intensities of exercise will provide additional benefit beyond that induced by submaximal/maximal exercise training, at least in terms of the PGC-1α mediated muscle adaptive response [37]. Given the current popularity of high-intensity interval training (HIT), and the potential safety [38] and adherence [39], [40] concerns often speculated with prescribing HIT in clinical populations, the lower increases in PGC-1α mRNA following intervals at 133% compared to 100% of peak aerobic power suggest that these concerns may outweigh the potential benefits associated with supramaximal HIT protocols. It is important to note that our results do not exclude the possibility for supramaximal HIIE to induce mitochondrial biogenesis to a greater extent following chronic exercise training in comparison to maximal or submaximal HIIE. Therefore, our results highlight the need for future studies designed to examine the impact of exercise intensity post-training interventions, while controlling for exercise volume, on mitochondrial adaptation and metabolic health.

### Relationship Between PGC-1α Expression and Muscle Activation

Whether differences in muscle fibre recruitment are responsible for the observed “intensity-dependent” effects of exercise has recently received increasing attention [14], [22], [23], [41]. When intensity-dependent changes in either gene expression or intramuscular signalling are examined in muscle homogenates there are two possible interpretations for an observed increase: 1) higher intensities of exercise cause greater intrinsic adaptations in a specific pool of muscle fibres (e.g., type II fibres during high intensity exercise) or 2) greater changes following high intensity exercise reflect similar adaptation in a greater number of fibres. In support of the latter, our data indicate that the greater increase in PGC-1α gene expression observed in the 100% compared to the 73% condition was directly proportional to the observed increase in muscle activation in the 100% condition (Figure 2B). This result suggests that the greater increases in both PGC-1α [10], [14], [15] and intracellular signalling directed towards mitochondrial biogenesis [9]–[12] observed previously may reflect greater fibre recruitment during higher intensities of submaximal exercise rather than an intensity specific effect *per se*. Interestingly, when expressed relative to muscle activation, the increase in PGC-1α gene expression following exercise at 133% of peak aerobic power was significantly lower than both the 73 and 100% conditions. This disassociation of PGC-1α gene expression from muscle activation supports the contention that the activation of PGC-1α is being blunted during supramaximal exercise. Due to the design of the present study, which aimed to examine changes in gene expression following an acute bout of exercise, we did not take biopsies immediately following exercise and are therefore unable to comment with certainty on the exact signalling mechanisms responsible for the blunted increase in PGC-1α mRNA in the 133% condition. Our surprising result highlights the need for studies comparing the impact of submaximal, maximal and supramaximal exercise of matched total exercise volume on activation of PGC-1α transcription.

### Exercise Intensity-Independent Gene Expression of PGC-1 α Regulators

Our results suggest that signals that activate PGC-1α transcription (i.e. AMPK and CAMK/p38 MAPK signalling pathways) may not respond in an exercise intensity-dependent fashion to supramaximal exercise. Alternatively, it is possible that factors responsible for inhibition of PGC-1α such as RIP140 [42] or GCN5 [35] may serve to inhibit PGC-1α transcriptional activation in response to supramaximal exercise. The transcription co-repressor RIP140 is proposed to inhibit mitochondrial biogenesis by directly interacting with and inhibiting PGC-1α activity [43], as well as by acting as a scaffolding protein for histone deacetylases involved in chromatin remodelling [44]. Interestingly, recent work has indicated that RIP140 protein may be rapidly upregulated in response to high-intensity exercise [45]. There is controversy as to whether changes in RIP140 protein content may play a role in regulating exercise-induced mitochondrial biogenesis [45]–[47]. However, the above findings are in agreement with the results of the current study, and others [46], that RIP140 gene expression is increased three hours post-exercise (in this case independent of exercise intensity). GCN5 is a protein acetylase that negatively regulates PGC-1α activity [35], functioning in opposition to SIRT1. The current observation that GCN5 gene expression is unaltered following acute exercise, combined with the previous observation that GCN5 appears to be shuttled out of the nucleus following acute exercise [48], suggest that altered expression of GCN5 is not critical in the post exercise regulation of PGC-1 α activity. Future research should focus on transient post-translational regulation of GCN5 activity and whether increases in RIP140 expression are relevant to the regulation of mitochondrial biogenesis in human skeletal muscle.

### Changes in Gene Expression of SIRT1 Regulators

The observation that SIRT1 mRNA expression is modestly increased following all three intensities of exercise examined in the current study demonstrates that SIRT1 gene expression can be induced by short duration, interval exercise in addition to long duration endurance exercise [19], [20]. Further, we have demonstrated for the first time that increases in SIRT1 mRNA following an acute bout of exercise appear to be independent of exercise intensity. While the mechanisms underlying this intensity-independent increase in SIRT1 expression are unclear, a recent report has suggested that mechanical stress induces SIRT1 expression via EGR1 [18]. Alternatively, SIRT1 expression is also controlled by E2F transcription factor 1 (E2F1) [49] and by p53 activity [50]; the latter of which is activated in response to exercise [41]. Whether these transcription factors are activated differentially by higher intensities of exercise is unknown, however, p53 acetylation status appears to be altered similarly by HIT and endurance exercise [41]. It is also possible that factors responsible for controlling SIRT1 expression are activated by intensity-independent stress (e.g., mechanical stretch) and contribute to the increases in SIRT1 expression observed in past reports [19], [20] and the current study.

SIRT1 gene expression is consistently observed to increase following an acute bout of exercise, while SIRT1 protein content following exercise training has been observed to increase [7], [51], [52], decrease [8], [53], [54] and remain unchanged [55]. Because of the observed increase in SIRT1 mRNA following exercise, we also examined proposed regulators of SIRT1 gene expression: EGR1 and p53. EGR1 is a zinc finger transcription factor involved in the regulation of cell growth, proliferation, differentiation, and apoptosis [56]. In agreement with our findings, several studies have reported increases in EGR1 gene expression in skeletal muscle following mechanical stress [18], [57], [58]. Resistance exercise in young and old men have inverse results, with young men exhibiting a decrease in EGR1 expression while older adults had an increase following an acute bout of resistance exercise [57]. Altogether, it appears that EGR1 gene expression is regulated by exercise, and results from the current study indicate that it is upregulated to a greater extent at supramaximal exercise intensities. Similar to EGR1, the tumor suppressor p53 was also elevated at all three intensities of HIIE. In skeletal muscle, p53 is responsive to cellular stress and is a key regulator of cellular metabolism [59]. While no other studies have examined changes in skeletal muscle p53 mRNA in response to exercise, one study examined changes in p53 protein content in response to an acute bout of maximally activated eccentric contractions. Interestingly, six hours post-exercise p53 protein content increased, and this was preceded by an increase in nuclear p53 one hour post-exercise [60]. Thus, while a variety of factors may be influencing changes in SIRT1 protein content following chronic contractile activity and exercise training, it would appear that factors involved in controlling SIRT1 expression are acutely activated by exercise.

### Limitations and Future Directions

The precise intramuscular mechanisms underlying the dissociation of PGC-1α expression from exercise intensity and muscle activation following supramaximal exercise cannot be ascertained in the present study design. Due to our primary objective of examining changes in post exercise gene expression (typically observed three hours post-exercise) along with muscle activation using a crossover study design in humans, the number of biopsies was limiting. However, our results will help guide further work on intensity-dependent regulation of PGC-1α and SIRT1 gene expression. Of specific interest are the changes in intracellular signalling and/or PGC-1α inhibitors that may explain the apparent blunting of PGC-1α activation following supramaximal interval exercise. An additional extension of the current work would be to examine whether the lesser increase in PGC-1α expression following an acute bout of supramaximal exercise is reflected in altered upregulation of mitochondrial content following a training intervention. This was also outside the scope of the present study, but studies examining whether supramaximal intensities of exercise induce additional adaptation beyond that observed following submaximal/maximal exercise represent a critical area for future work.

Finally, we have estimated muscle activation using surface EMG, and while this technique provides a continuous, non-invasive means of estimating total fibre recruitment, its ability to provide information on recruitment patterns of specific fibre types is controversial [61], [62]. Future work combining exercise EMG derived estimates of muscle activation and fibre type recruitment (e.g. wavelet analysis) with post exercise immunohistochemistry may help provide further information on the impact of exercise intensity of fibre specific adaptation. Further, recent developments in immunofluorescence analysis of muscle sections [63] may prove valuable for studies attempting to examine semi-quantitative fibre specific adaptations to different intensities of exercise training.

### Conclusions

The current study examined the impact of altered exercise intensity, while maintaining a fixed exercise volume, on post-exercise gene expression of PGC-1α and its regulators. While PGC-1α mRNA expression increased to a greater extent following interval exercise at 100% compared to 73%, this apparent intensity effect did not extend to supramaximal intervals performed at 133% of peak aerobic power. The lower expression of PGC-1α relative to EMG derived estimates of muscle activation suggests that signalling mediated activation of PGC-1α may be blunted during supramaximal intervals. We also provide novel data that SIRT1 and RIP140 expression is induced in an intensity-independent fashion following all intensities of exercise. These results question whether supramaximal interval training will provide additional benefit beyond that obtained from submaximal and maximal exercise.
